# Influence of Aflibercept on Choroidal Blood Flow and Thickness in Branch Retinal Vein Occlusion: A Six-Month Follow-Up Study

**DOI:** 10.3390/diagnostics14222484

**Published:** 2024-11-07

**Authors:** Ryuya Hashimoto, Kenichiro Aso, Keisuke Yata, Kazufumi Tanaka, Naoki Fujioka, Ryo Yamazaki, Serika Moriyama, Juri Kawamura, Asato Hirota, Takatoshi Maeno

**Affiliations:** Department of Ophthalmology, Toho University Sakura Medical Center, 564-1, Shimoshizu, Sakura City 285-8741, Japan

**Keywords:** aflibercept, anti-VEGF drugs, branch retinal vein occlusion, choroidal blood flow, laser speckle flowgraphy

## Abstract

Background/Objectives: We intended to investigate choroidal blood flow (CBF) and choroidal thickness (CT) alternations in treatment-naive eyes with non-ischemic branch retinal vein occlusion (BRVO) following intravitreal aflibercept injection (IVA). Methods: Twenty eyes of 20 patients with treatment-naive non-ischemic BRVO, treated with IVA 1+ pro re nata, were included in this study. In the BRVO eyes, CBF and CT were measured in the occlusive region, subfovea, as well as the non-occlusive region, via laser speckle flowgraphy (LSFG) and enhanced depth-imaging optical coherence tomography over a 6-month follow-up period. CBF was analyzed via the mean blur rate using LSFG analysis software (version 3.10.0). Results: CT showed significant reductions in both the occlusive and subfoveal region at 1 week and 1 month after treatment (both *p* < 0.05). CBF was significantly decreased in the subfovea and the non-occlusive region at 1 week and 1 month from baseline, respectively (both *p* < 0.05). The mean number of IVA injections during the 6-month period was 1.95 ± 0.6. Conclusions: Aflibercept treatment reduced CBF and CT in addition to a decrease in retinal thickness. These changes at each region might be associated with the improvement of macular edema in BRVO eyes.

## 1. Introduction

Branch retinal vein occlusion (BRVO) represents a common retinal vascular disorder that leads to macular edema (ME) [1] and subretinal hemorrhage [2,3], resulting in disruption of foveal photoreceptors. ME is one of the main reasons for impairment of visual acuity (VA) in patients with BRVO. The pathogenesis of ME in BRVO eyes has been hypothesized to be caused by breakdown of the blood–retinal barrier due to damage to the tight junction of endothelial cells [4], as well as hypervascular permeability caused by vascular endothelial growth factor (VEGF) produced by the ischemic retina [5]. Clinicians have multiple options for treating ME in the setting of BRVO [1]. The VEGF pathway has proven to be a crucial therapeutic target, with several inhibitors (ranibizumab, aflibercept, and faricimab) demonstrating clinical success in reducing BRVO-associated edema [6,7,8,9,10].

While overexpression of VEGF causes ME, physiologically active levels of VEGF play an important role in the development and maintenance of choroidal vascular tissue [11]. The choroid has been reported to be one of the most highly vascularized tissues of the body [12]. Choroidal blood flow (CBF) is important in terms of supplying oxygen and nutrients to the outer retina, including retinal pigment epithelial (RPE) cells, as well as removing waste products from the RPE cells [12,13]. Furthermore, CBF and choroidal thickness (CT) have been reported to be related to the luminal area in the choroidal tissues [14], and severe choroidal attenuation and vasculature loss frequently result in photoreceptor degeneration [12]. Considering these clinical findings, it is important to identify whether CBF and CT are influenced by anti-VEGF therapy in BRVO eyes. Previous studies have reported changes in ocular circulation in BRVO eyes after injection of anti-VEGF agents [15,16,17,18,19,20,21]. However, the longitudinal changes in CBF and CT following IVA therapy remain unexplored in treatment-naive BRVO, particularly regarding regional differences. We monitored these choroidal measurements in occlusive and non-occlusive regions throughout a 6-month post-IVA period.

## 2. Materials and Methods

### 2.1. Participants

Twenty treatment-naive eyes with non-ischemic BRVO from 20 consecutive subjects (8 males and 12 females, mean age 69.6 ± 9.3 years) were enrolled.

This study was performed at our hospital between March 2015 and November 2016. The research protocol followed the principles outlined in the Declaration of Helsinki and received approval from the Ethics Committee at Toho University Sakura Medical Center (approval number: 2015056, 28 December 2015).

Inclusion criteria for treatment-naive non-ischemic BRVO were macular edema onset within 12 weeks, central foveal thickness (CFT) exceeding 300 μm as measured by optical coherence tomography (Spectralis OCT^®^, Heidelberg Engineering Inc., Heidelberg, Germany), and symptom duration of less than 6 months before initial examination.

Retinal specialists diagnosed BRVO using fundus examination and fluorescein angiography. Cases were classified as either major BRVO or macular BRVO and analyzed accordingly [22].

Exclusion criteria encompassed glaucoma, vitreoretinal disorders, prior vitreous surgery or laser photocoagulation, previous anti-VEGF or steroid treatment, use of anticoagulants/antiplatelets/corticosteroids, and high myopia (>−6 diopters).

### 2.2. Therapy for Macular Edema Related to Branch Retinal Vein Occlusion and Follow-Up

Aflibercept (Eylea^®^; Regeneron Pharmaceuticals, Inc., Tarrytown, NY, USA and Bayer Healthcare Pharmaceuticals, Berlin, Germany) (0.5 mg/0.05 mL) was injected into the vitreous body using a 30-gauge needle approximately 3.5–4.0 mm from the limbus after placement of 0.4% oxybuprocaine drops (Benoxyl; Santen Pharmaceutical Co., Ltd., Osaka, Japan). Each eye initially received one injection of aflibercept, followed by a pro re nata (PRN) regimen. Additional injections were administered when CFT exceeded 300 µm with persistent intraretinal fluid on OCT. Recurrent ME was treated with aflibercept injection at a minimum interval of 1 month from the previous treatment.

### 2.3. Eye Examinations

All patients received ocular examinations every month after the IVA injection during the 6-month follow-up period, and the results at day 1; week 1; and months 1, 2, 3, 4, 5, and 6 were analyzed. Our examination protocol consisted of functional and structural assessments. LogMAR visual acuity was calculated from decimal measurements using Landolt C charts. Ocular imaging included slit-lamp examination, fundus photography, both conventional and enhanced depth imaging (EDI)-OCT, and LSFG for blood flow analysis.

### 2.4. Assessment of Choroidal and Retinal Thickness

**Figure 1:**
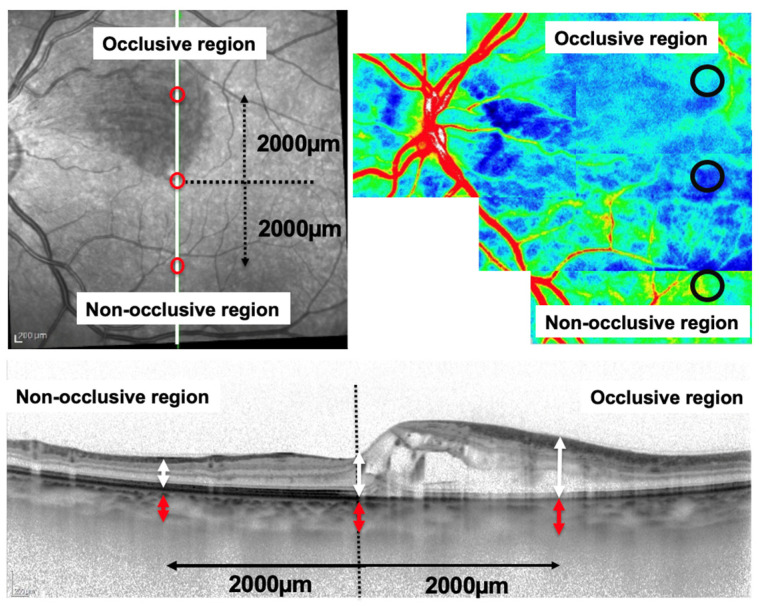
Evaluation of retinal and choroidal parameters using enhanced depth-imaging optical coherence tomography and laser speckle flowgraphy in eyes with branch retinal vein occlusion.

CT measurements were obtained using EDI-OCT [23,24,25,26,27]. The retinal thickness and CT of the central fovea and subfovea, the occlusive and the non-occlusive regions, were measured using vertical scan images by three independent examiners (J.K., and A.H.) (Figure 1, top left).

As described in our previous studies [28,29], the occlusive area was defined as the arteriovenous crossing located 2000 µm superior and 2000 µm inferior to the fovea.

EDI-OCT scans for CT analysis were obtained during a controlled time frame (12:00–15:00) to minimize the influence of diurnal variations [30], as shown in Figure 1 (bottom).

### 2.5. Assessment of Choroidal Blood Flow

This study employed the LSFG-NAVI™ system (Softcare Co., Ltd., Fukuoka, Japan) to quantify CBF in patients with treatment-naive non-ischemic BRVO. LSFG is a non-invasive, validated method for evaluating ocular circulation, with high reproducibility in clinical research [14,31,32,33,34,35,36,37,38,39]. The assessment protocol used in this study follows established methodologies [40,41], with the mean blur rate (MBR) serving as a marker for blood flow [42].

CBF was measured in three predefined regions: the occlusive region, the subfoveal area, and the non-occlusive region. Measurement circles were manually aligned using LSFG composite images and fundus photographs to ensure precision, as described in our previous studies [28,29] (Figure 1, top left).

MBR measurements were obtained in triplicate at each time point using LSFG and averaged for statistical analysis.

To track longitudinal changes in CBF, MBR values were calculated for each region using LSFG Analyzer software (Version 3.10.0, Softcare Co., Ltd., Fukuoka, Japan). These values were expressed as a percentage relative to the baseline, providing a comprehensive metric for evaluating temporal changes in ocular blood flow throughout the study period.

### 2.6. Ocular Hemodynamics

We measured intraocular pressure (IOP) using a non-contact tonometer (Canon TX-F; Canon Inc., Tokyo, Japan, and systolic blood pressure (SBP) and diastolic blood pressure (DBP) using an automated sphygmomanometer (HBP-9020; OMRON Healthcare, Kyoto, Japan) in the sitting position at the same time as measuring CBF, given the linear relationship between CBF and ocular perfusion pressure (OPP) [43].

Mean blood pressure (MBP) and OPP were determined according to previously reported equations [44,45,46].

### 2.7. Statistical Analysis

Data are presented as mean ± standard deviation (SD). Data normality was evaluated using the Shapiro–Wilk test. To analyze temporal changes in logMAR VA, retinal thickness, CT, and CBF from baseline, we employed one-way repeated-measures analysis of variance (ANOVA) with post hoc Bonferroni adjustment. Regional differences in retinal thickness, CT, and CBF were assessed using repeated-measures ANOVA. Statistical analyses were performed using GraphPad Prism version 9 (GraphPad Software, San Diego, CA, USA), with *p* < 0.05 considered significant.

## 3. Results

### 3.1. Clinical and Laboratory Characteristics of Patients

Table 1 shows the baseline characteristics of the 20 enrolled patients (8 men, 12 women; mean age, 69.6 ± 9.3 years). The study included 9 eyes with major BRVO and 11 with macular BRVO. The mean arm-to-retina circulation time was 18.9 ± 4.4 s. More patients had a superior (*n* = 13) than inferior (*n* = 7) occlusive region. Concurrent systemic diseases included hypertension (11/20, 55.0%), hyperlipidemia (8/20, 40%), and type 2 diabetes mellitus (1/20, 5.0%).

The mean number of additional aflibercept injections over 6 months was 1.95 ± 0.60 (range, 1–3).

The mean baseline logMAR VA was 0.50 ± 0.33. Although logMAR VA at 1 day after IVA injection (0.43 ± 0.30) was not significantly different (*p* = 1.00), it improved to 0.23 ± 0.22, 0.18 ± 0.25, 0.17 ± 0.24, 0.19 ± 0.21, 0.15 ± 0.24, 0.09 ± 0.21, and 0.10 ± 0.17 at day 7 and months 1, 2, 3, 4, 5, and 6 after the IVA injection, respectively (all *p* < 0.05 vs. baseline).

### 3.2. Regional Distribution of Retinal and Choroidal Parameters Before Intravitreal Aflibercept Injections in Affected Eyes

**Figure 2:**
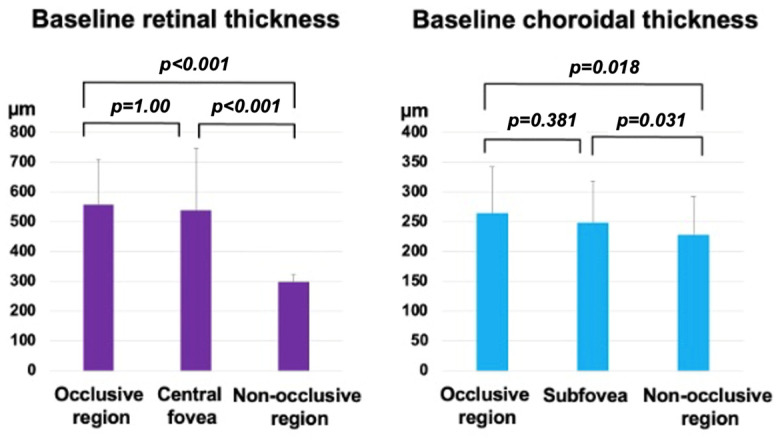
Baseline mean retinal and choroidal thicknesses in branch retinal vein occlusion eyes.

Baseline morphometric analysis demonstrated regional differences in retinal thickness (Figure 2, left). The occlusive region exhibited significant thickening (559 ± 150 µm) relative to the non-occlusive region (300 ± 23.5 µm; *p* < 0.001). CFT (539 ± 206 µm) did not differ significantly from that in the occlusive region (*p* = 1.00). CT also showed regional differences; the values were significantly greater in the occlusive region (265 ± 77.4 µm) than in the non-occlusive region (229 ± 63.1 µm; *p* = 0.018) (Figure 2, Right). Subfoveal CT (249 ± 69.9 µm) showed no significant difference from the occlusive region (*p* = 0.381). Correlation analysis indicated a trend toward an association between CT and retinal thickness in the occlusive region (r = 0.427, *p* = 0.061, Pearson’s correlation analysis).

### 3.3. Changes in Retinal and Choroidal Parameters Following Intravitreal Aflibercept Injections in Affected Eyes

**Figure 3:**
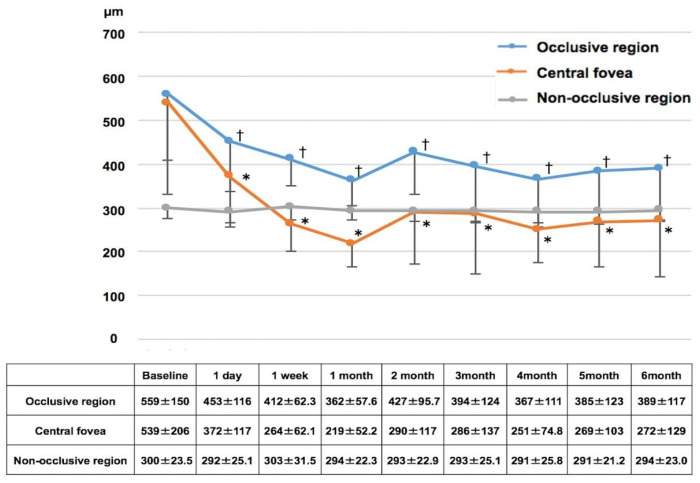
Changes in retinal thickness following aflibercept treatment in branch retinal vein occlusion eyes over 6 months. † *p* < 0.05 versus baseline (occlusive region), * *p* < 0.05 versus baseline (central fovea). (All *p* values adjusted with Bonferroni correction).

Sequential analysis of retinal thickness (Figure 3) showed significant reductions from baseline in both CFT (539 ± 206 µm) and occlusive region measurements (559 ± 150 µm) at all post-treatment time points (all *p* < 0.05). The non-occlusive region showed no significant changes throughout the follow-up period.

**Figure 4:**
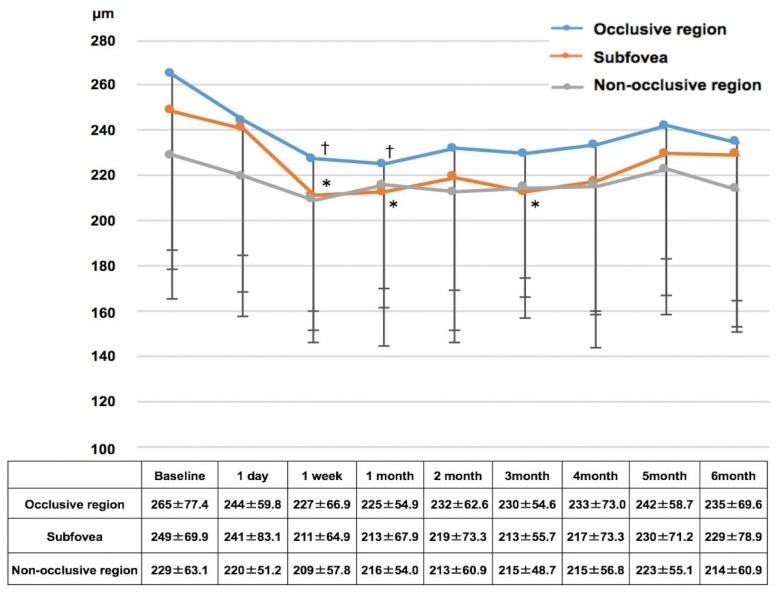
Changes in choroidal thickness following aflibercept treatment in branch retinal vein occlusion eyes over 6 months. † *p* < 0.05 versus baseline (occlusive region), * *p* < 0.05 versus baseline (subfovea). (All *p* values adjusted with Bonferroni correction).

CT measurements (Figure 4) showed significant decreases from baseline in the subfoveal area (249 ± 69.9 µm) at 1 week, 1 month, and 3 months after treatment (all *p* < 0.05). The occlusive region showed significant reductions from baseline values (265 ± 77.4 µm) at 1 week and 1 month (all *p* < 0.05). The non-occlusive region remained stable throughout the follow-up period.

### 3.4. Changes in Choroidal Blood Flow Following Aflibercept Treatment in BRVO Eyes over 6 Months

**Figure 5:**
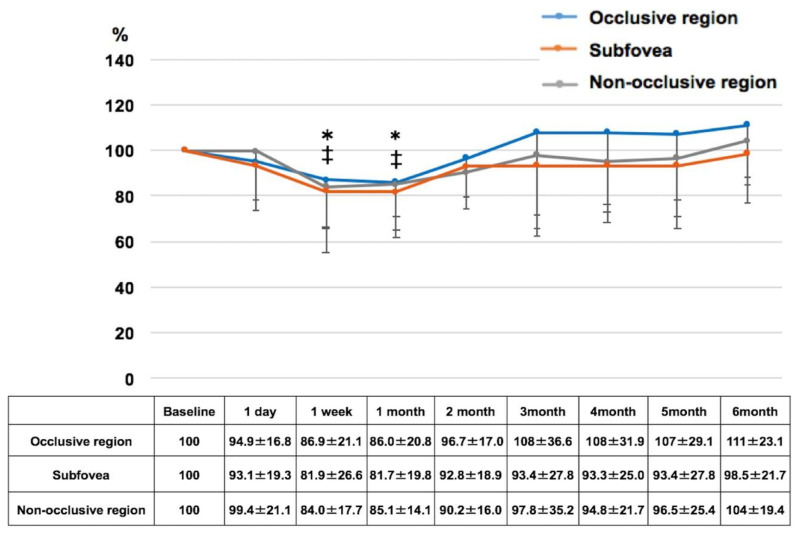
Changes in choroidal blood flow following aflibercept treatment in branch retinal vein occlusion eyes over 6 months. ‡ *p* < 0.05 versus baseline (non-occlusive region), * *p* < 0.05 versus baseline (subfovea). All *p* values adjusted with Bonferroni correction.

The change in the mean CBF (%) in each region in BRVO eyes after IVA injection over a 6-month period is shown in Figure 5. The CBF values at each of these regions were as follows: 1-day (94.9 ± 16.8/93.1 ± 19.3/99.4 ± 21.1), 1-week (86.9 ± 21.1/81.9 ± 26.6/84.0 ± 17.7), 1-month (86.0 ± 20.8/81.7 ± 19.8/85.1 ± 14.1), 2-month (96.7 ± 17.0/92.8 ± 18.9/90.2 ± 16.0), 3-month (108 ± 36.6/93.4 ± 27.8/97.8 ± 35.2), 4-month (108 ± 31.9/93.3 ± 25.0/94.8 ± 21.7), 5-month (107 ± 29.1/93.4 ± 27.8/96.5 ± 25.4), and 6-month (111 ± 23.1/98.5 ± 21.7/104 ± 19.4) follow-up time points. The mean CBF values in the subfovea (81.9 ± 26.6 and 81.7 ± 19.8) and non-occlusive region (84.0 ± 17.7 and 85.1 ± 14.1) were significantly reduced at 1 week and 1 month after injection of IVA (*p* < 0.05, respectively). There were no significant changes in CBF in the occlusive region during the follow-up period.

### 3.5. Hemodynamics

The SBP/DBP (mmHg) did not change significantly from baseline (143 ± 17.8/81.2 ± 10.1) at the 1-day (143 ± 16.9/81.5 ± 16.3), 1-week (139 ± 16.9/80.7 ± 11.2), 1-month (142 ± 20.7/79.1 ± 12.8), 2-month (145 ± 24.7/80.6 ± 15.7), 3-month (145 ± 23.3/80.1 ± 11.4), 4-month (144 ± 19.1/79.7 ± 12.4), 5-month (146 ± 18.0/80.1 ± 9.1), or 6-month (147 ± 19.0/82.1 ± 13.3) follow-up time points after IVA injections.

The MBP (mmHg) did not change significantly from baseline (102 ± 11.5) at the 1-day (102 ± 15.4), 1-week (100 ± 11.9), 1-month (100 ± 14.4), 2-month (102 ± 17.6), 3-month (102 ± 14.0), 4-month (101 ± 13.0), 5-month (102 ± 9.1), or 6-month (103 ± 11.0) follow-up time points after IVA injections.

The mean OPP (mmHg) was 54.8 ± 7.3 at baseline, and did not change significantly from baseline at the 1-day (56.5 ± 9.7), 1-week (55.0 ± 7.7), 1-month (54.6 ± 8.7), 2-month (55.4 ± 10.1), 3-month (54.5 ± 8.2), 4-month (54.9 ± 7.8), 5-month (55.5 ± 5.3), or 6-month (56.4 ± 8.0) follow-up time points.

### 3.6. Safety

There were no serious adverse effects related to IVA injections in any patient during the 6-month follow-up period.

## 4. Discussion

This study evaluated changes in CBF and CT following IVA treatment in treatment-naive non-ischemic BRVO over 6 months. Significant reductions in CT were observed in both subfoveal and occlusive regions after IVA injection, along with decreased retinal thickness. CBF showed significant temporary decreases in the subfoveal and non-occlusive regions at 1 week and 1 month after treatment.

Regarding the relationship between choroidal circulation and BRVO, CBF in BRVO eyes was previously reported to be elevated to compensate for the ischemic retina [47]. Several other authors have reported changes in retinal [16,17] and choroidal blood flow [19] in BRVO eyes after injection of anti-VEGF agents. Nagaoka and associates previously reported that one intravitreal bevacizumab (IVB) injection had little effect on the retinal blood flow in patients with ME secondary to BRVO during a 3-month follow-up period [16]. In contrast, Fukami et al. demonstrated reduced retinal blood flow in BRVO eyes at 1 week and 1 month after ranibizumab injection [20]. In view of the change in CBF after anti-VEGF treatment in some patients with BRVO, but not in others, it has been hypothesized that there is a “good therapeutic response” group and “poor therapeutic response” group [19]. However, it remains controversial whether ocular blood flow significantly decreases or increases after anti-VEGF therapy in BRVO eyes.

IVA injections are known to decrease the concentration of aqueous VEGF in non-vitrectomized macaque eyes for 6 weeks. Furthermore, IVA injections have been shown to reduce aqueous VEGF levels below the limit of detection for longer periods than IVR [48]. Aflibercept has been reported to penetrate through the highly polarized RPE layers in vitro, although aflibercept showed the lowest ability among the three anti-VEGF agents (ranibizumab, aflibercept, and bevacizumab) to permeate though the RPE layer [49]. Generally, the choriocapillaris of the eye has been shown to be vulnerable to inhibition of VEGF [50]. Although reduced fenestrations of the choriocapillaris and vessel occlusion were induced temporarily after intravitreal injection of anti-VEGF agents in primate eyes, the fenestrations gradually started to increase after 7 days [51]. In the current study, the mean CBF in the subfovea and non-occlusive regions and CT in the occlusive and subfovea regions in BRVO eyes decreased significantly by 1 week after IVA injections. However, CT and CBF in these regions tended to recover back to baseline levels by 2 months. Considering these clinical findings, a significant decrease in intraocular (vitreous fluids or anterior chamber) VEGF concentration via IVA injections might strongly and temporally affect the decrease in CBF and CT in BRVO eyes.

In terms of systemic effects, plasma and serum VEGF levels following anti-VEGF therapy have been investigated in age-related macular degeneration [52,53,54]. Wang and associates reported that aflibercept significantly reduced the serum and plasma VEGF concentrations by 1 week and 1 month after IVA injection, although VEGF concentrations returned to baseline levels after 2 months [52]. Although we did not investigate the CBF in the non-injected eyes after IVA injections, it is possible that aflibercept might escape into the systemic blood circulation and have a slight effect on the choroidal circulation, in addition to its localized action. Further studies are needed to identify the mechanism.

In this study, baseline CT was significantly greater in the occlusive region compared with the non-occlusive region in BRVO eyes, which is in agreement with previous studies [28,29,55]. VEGF increases vessel permeability and stimulates nitric oxide release [56], leading to retinal arteriolar dilation [57,58]. Vitreous VEGF levels are elevated in BRVO compared with control eyes [59,60], affecting both outer retinal and choroidal vasculature in ischemic areas. (i.e., occlusive regions) [11,61]. Considering the above reports and that the luminal area in the choroid was significantly and positively correlated with the CT [14], it is plausible that the retinal and choroidal thickness in the occlusive regions might be thicker than that in the non-occlusive regions.

The present study has several limitations. First, the results were obtained from a small number of participants and over a relatively short follow-up period. Further validation studies with more subjects and longer observational periods are needed to evaluate the time course of CBF after anti-VEGF treatments. Second, there were no significant differences in CBF in the occlusive region after IVA injections, whereas the mean CBF values in the subfovea and non-occlusive regions were significantly decreased by 1 week and 1 month after IVA injection. Further studies are required in order to elucidate the underlying mechanism. Third, we did not measure the serum or plasma concentrations of VEGF in the studied subjects. In order to elucidate whether systemic changes in VEGF concentration influence the CBF in the affected and fellow eyes, serum and plasma VEGF levels should be examined in addition to CBF in the non-injected fellow eyes. Furthermore, VEGF levels in the aqueous or vitreous fluid need to be measured to validate the regional changes in VEGF after anti-VEGF treatments. Moreover, we acknowledge the absence of statistical power calculations as a limitation. Given our limited sample size, this may impact the generalizability and reliability of our findings. Future studies will include larger cohorts and statistical power analysis to validate the efficacy of anti-VEGF treatments and the strength of our conclusions regarding the effectiveness of anti-VEGF treatments.

## 5. Conclusions

In conclusion, the CBF values in the subfovea and non-occlusive region were significantly decreased by 1 week and 1 month after aflibercept therapy, as compared to baseline, during the 6-month follow-up period. Aflibercept treatment reduced CBF and CT in addition to a decrease in retinal thickness. These changes at each region might be associated with an improvement of macular edema in treatment-naïve BRVO eyes.

## Figures and Tables

**Table 1:** Baseline characteristics of study participants.

Patients (*n*)	20
Men:women	8:12
Age (years)	69.6 ± 9.3
Major BRVO:macular BRVO	9:11
Superior:inferior (occlusive region)	13:7
Axial length (mm)	23.9 ± 1.4
Systolic blood pressure (mmHg)	143 ± 17.7
Diastolic blood pressure (mmHg)	81.2 ± 10.9
Mean blood pressure (mmHg)	102 ± 11.5
Ocular perfusion pressure (mmHg)	54.8 ± 7.3
Arm to retina circulation time (seconds)	18.9 ± 4.4
Hypertension (%)	11/20 (55.0)
Hyperlipidemia (%)	8/20 (40.0)
Type 2 diabetes mellitus (%)	1/20 (5.0)
Administration of ARB (%)	4/20 (20.0)
Administration of CCB (%)	6/20 (30.0)
Administration of statin (%)	5/20 (20.0)
Administration of hypoglycemic agents (%)	1/20 (5.0)
Triglyceride (mg/dL)	138 ± 77.1
HDL cholesterol (mg/dL)	56.3 ± 13.6
LDL cholesterol (mg/dL)	128 ± 30.9
Fasting plasma glucose (mg/dL)	110 ± 37.1
Hemoglobin A1c (%)	5.8 ± 0.3
eGFR (mL/minutes per 1.73 m^2^)	72.3 ± 14.6
Red blood cells (×10^6^ μL)	4.6 ± 0.4
Hemoglobin (g/dL)	13.8 ± 1.3
Hematocrit (%)	40.9 ± 3.5

Data are presented as mean ± SD.

## Data Availability

Data are available from the corresponding author upon reasonable request.
